# Small airways dysfunction: The importance of utilising Z-scores to define MMEF abnormalities in clinical practice

**DOI:** 10.1016/j.heliyon.2023.e20744

**Published:** 2023-10-06

**Authors:** Mohammed A. Almeshari, Nowaf Y. Alobaidi, Elizabeth Sapey, Robert A. Stockley, James A. Stockley

**Affiliations:** a Rehabilitation Health Sciences Department, College of Applied Medical Sciences, King Saud University, Riyadh, Saudi Arabia; b Institute of Inflammation and Ageing, University of Birmingham, Edgbaston, Birmingham, UK; c Respiratory Therapy Department, College of Applied Medical Sciences, King Saud Bin Abdul-Aziz University for Health Sciences, Al Ahsa, Saudi Arabia; d Department of Lung Function and Sleep, University Hospitals Birmingham NHS Foundation Trust, Edgbaston, Birmingham, B15 2GW, UK

**Keywords:** Spirometry, Z-score, Standardised residual, Small airways, FEF25-75, MMEF

## Abstract

**Background:**

The small airways comprise the largest cross-sectional area of the lungs, however, assessing and reporting abnormalities for this region of the bronchial tree has been practically and scientifically uncertain.

**Methods:**

Using routinely collected spirometry data for patients with either asthma or COPD, the accuracy of % predicted values for defining small airways dysfunction was assessed. A z-score of ≤ −1.645 of the maximal-mid expiratory flow (MMEF) was used as the gold standard for defining abnormality in the small airways.

**Results:**

Records of 3396 patients were included in the analysis. The false positive (FP) rates were 24.6 %, 16.1 %, 11.5 %, or 7.9 % when the % predicted value of 80 %, 70 %, 65 %, or 60 % were used, respectively. Sex, age, and BMI were associated with FP rates. Males were more likely to be categorised as FP with odds ratio (OR) between 1.10 and 1.49 across % predicted groups. Age was associated with FP rates with an OR between 1.01 and 1.08. The BMI was also associated with FP rates with an OR of 1.03 across all % predicted groups. Assessing the association of age groups with FP rate showed that those above 60 years old were more likely to be categorised as FP with an OR between 1.23 and 73.2 compared to those less than 30 years old.

**Conclusion:**

When assessing the small airways in clinical practice or for scientific purposes, the % predicted values overestimate the actual impairment leading to FP interpretation. Utilising z-score values are recommended to assess the small airways using the spirometric index, MMEF.

## 1 Background

Spirometry is the most commonly used, objective measure for lung function in clinical practice. It is non-invasive, widely available, can be portable, and is relatively easy to operate and interpret. The forced vital capacity (FVC) manoeuvre generates a number of integral parameters, including the forced expired volume in 1st second (FEV_1_) and a series of less commonly reported volume and flow indices. The maximal mid-expiratory flow (MMEF) was first introduced by Lueallan and Fowler in 1955 [1]. The MMEF is also known as the forced expiratory flow rate between the 25th and 75th percentiles (FEF_25-75_) of FVC. It is considered a measure of the central to small airways determined over the middle phase of the FVC manoeuvre.

The small airways comprise more than 98 % of the lungs cross-sectional area [2] and recent evidence suggest their functional impairment occurs in the early phase of chronic respiratory diseases such as asthma and Chronic Obstructive Pulmonary Disease (COPD) and worsens with disease progression, being highly correlated with disease severity [[3], [4], [5], [6], [7]].

Assessing and reporting the MMEF in routine clinical practice has been practically and scientifically uncertain [4,[8], [9], [10], [11]]. The MMEF has been described as a highly variable index, which may reflect effort dependence and heterogeneity in the small airways’ branching, lumen sizes, and the lack of cartilage to support the airways during expiration. However, a more recent study suggested that the variability of MMEF is comparable to that seen for FEV_1_ [12], making it more acceptable as a spirometric measure. In current guidelines, neither the appropriate evaluation nor the reporting of small airways function are well documented [13,14]. Despite great efforts in generating reference values for various spirometry indices (including MMEF), the reporting of indices are sub-optimal. Clinicians generally rely on % predicted to define abnormality and severity, based on reference ranges which are affected by age, sex, height and ethnicity. This persists even though evidence suggests that relying on such methodology alone may produce false positives (FP) or false negative (FN) results for impairment [15,16], especially at the extremes of age.

In the recent Association for Respiratory Technology and Physiology (ARTP) spirometry report, it was highlighted that the z-scores, also referred to as standardised residuals (SR), are more appropriate than using the fixed FEV_1_/FVC ratio or % predicted value for FEV_1_ to determine abnormality and severity [17].

It has been suggested that using z-scores is likely to reduce age and height bias [17]. Previous studies have suggested that the lower 5th percentile (the lower limit of normal (LLN); i.e., z-score: <–1.645) of the MMEF does not map to a consistency percent predicted cut off across populations or age groups [9,10]. The use of MMEF z-score in defining abnormality has not been previously compared to % predicted values in terms of sensitivity and specificity for defining abnormality. Therefore, the aim of this study was to evaluate the use of % predicted for evaluating MMEF abnormalities and identify any demographic features that may influence the % predicted values compared to the LLN as assessed by z-score in participants being evaluated for asthma or COPD.

## 2 Methods

### 2.1 Study design and setting

The data were obtained from the lung function department in a tertiary hospital in the West Midlands of the UK. The study and all study activities were approved by the Health Research Authority (HRA IRAS number 274729; REC Reference: 20/HRA/0203). The data were collected and compiled by the lung function department and anonymised prior to analysis.

### 2.2 Participants

Records of adults referred for routine lung function for either an asthma or COPD diagnosis, or for monitoring either disease and tested between 1st January 2016 and 30th April 2021 were used for the analysis.

### 2.3 Lung function testing

Participants were assessed using spirometry (Ultima PF Pulmonary Lung Function System (Medical Graphics, UK, Tewkesbury)), which was performed according to the ARTP/British Thoracic Society guidelines. The Global Lung Initiative (GLI) 2012 equation was used to calculate the % predicted and the z-score [18].

### 2.4 Statistical analysis and small airways dysfunction definition criteria

Due to the descriptive nature of this report, formal statistical analysis was not performed.

The 5th percentile (−1.645) of the z-scores for MMEF was used as the gold standard method for defining abnormality and, hence, small airways dysfunction (SAD). Although various % predicted cut-off points have been previously used to define MMEF abnormality [13,19]. The % predicted of <80 %, <70 %, <65 %, and <60 % were pragmatically chosen as the comparators. False positive was defined as % predicted less than the percentage threshold with a z-score > -1.645.

Logistic regression model was used to evaluate the predictors of FP and the odds ratios (OR) were reported.

## 3 Results

A total of 3396 participants were evaluated, of whom 1829 were being reviewed for asthma and 1567 for COPD. Table 1 provides a summary of the baseline data and demographics of the cohort.

**Table 1:** Demographics of the sample.

	Overall (N = 3396)	Asthma (N = 1829)	COPD (N = 1567)
Sex (Female)	1893 (55.7 %)	1124 (61.5 %)	769 (49.1 %)
Age (Years)	55.1 ± 17.1	47.4 ± 17.5	64.1 ± 11.1
(Range)	(18–90)	(18–89)	(31–90)
Age Groups			
<30	346 (10.2 %)	346 (18.9 %)	0 (0 %)
30-39	348 (10.2 %)	316 (17.3 %)	32 (2 %)
40-49	466 (13.7 %)	332 (18.2 %)	134 (8.6 %)
50-59	707 (20.8 %)	356 (19.5 %)	351 (22.4 %)
60+	1529 (45.0 %)	479 (26.2 %)	1050 (67 %)
BMI	28.9 ± 6.93	29.5 ± 6.84	28.2 ± 6.97
Ethnicity			
White	2814 (82.9 %)	1338 (73.2 %)	1476 (94.2 %)
BAME	582 (17.1 %)	491 (26.8 %)	91 (5.8 %)
MMEF z-score	−1.31 (−2.31 to −0.34)	−0.86 (−1.71 to −0.09)	−1.92 (−2.78 to −0.95)
MMEF % Predicted	62.9 (34.9–91.3)	78.1 (55.6–100)	41.8 (21.5–68.9)
FEV_1_/FVC z-score	−1.00 (−2.33 to −0.03)	−0.52 (−1.46 to 0.25)	−1.88 (−3.30 to −0.57)
FEV_1_/FVC ratio	73.0 (60.9–80.3)	77.9 (70.5–83.0)	64.0 (49.0–74.0)
80 % FP	837 (24.6 %)	468 (25.6 %)	369 (23.5 %)
70 % FP	546 (16.1 %)	258 (14.1 %)	288 (18.4 %)
65 % FP	392 (11.5 %)	161 (8.8 %)	231 (14.7 %)
60 % FP	268 (7.9 %)	97 (5.3 %)	171 (10.9 %)

Legend:

Values are reported as mean ± SD or median (Q1 to Q3) for continuous variables and n (%) for categorical variable.

**Abbreviations: BMI**, body mass index; **MMEF**, maximal mid-expiratory flow; **FEV**_**1**_**/FVC**, the ratio of the forced expiratory volume in the first second to the forced vital capacity; **BAME**, Black, Asian and Minority Ethnic; **FP**, False positive.

**Fig. 1:**
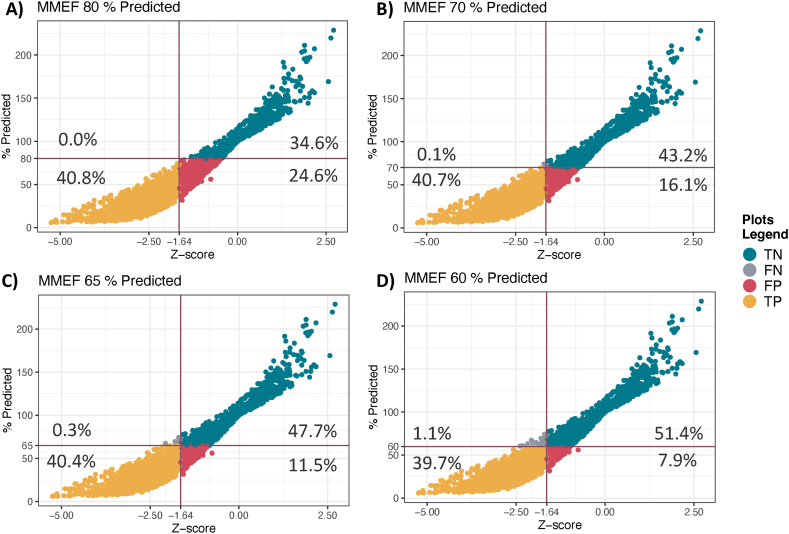
The proportion of false positive, false negative, true positive, true negative across multiple cut-off ranges for MMEF % predicted. **Legend:** The plots illustrate the proportions of false positives (FP), false negatives (FN), true positives (TP), and true negatives (TN). Using the 80 % predicted cut-off resulted in 24 % FP (A): 70 % predicted cut-off resulted in 16.1 % FP (B).:65 % predicted cut-off resulted in 11.5 % FP (C)and 60 % predicted cut-off resulted in 7.9 % FP (D). **Abbreviations:** MMEF, maximal mid-expiratory flow; FP, false positives; FN, false negatives; TP, true positives; TN, true negatives.

In both groups, the use of % predicted for the MMEF overestimated the LLN as seen in Table 1 and illustrated in Fig. 1 where the proportions of FP, true positive, true negative, and FN are plotted (A: 80 % predicted, B: 70 % predicted, C: 65 % predicted, D: 60 % predicted). Using the % predicted of MMEF, there were greater proportions of FP compared to FN.

The demographics of the FP and FN are reported in Table 2. There was equal sex representation. The FP group was older (mean age of 61.2 ± 15.8) than the other groups (55.1 ± 17.1). The FP cohort included 258 (47.3 %) patients with asthma, and 288 (52.7 %) patients with COPD. Overall, those above 60 years of age formed most of the false positive group n = 327 (59.9 %). The median FEV_1_/FVC z-score was within normal range (−1.08:IQR -1.46 to −0.67).

**Table 2:** Characteristic of the false positive and false negative groups.

	FP (n = 546; 16.1 %)	FN (n = 3; 0.1 %)
**Sex (Female)**	273 (50.0 %)	3 (100 %)
**Age (Years)**	61.2 ± 15.8	22.0 ± 5.29
**BMI**	30.0 ± 7.24	26.1 ± 1.63
**Age groups**		
<30	27 (4.9 %)	3 (100 %)
30-39	27 (4.9 %)	0 (0 %)
40-49	67 (12.3 %)	0 (0 %)
50-59	98 (17.9 %)	0 (0 %)
60+	327 (59.9 %)	0 (0 %)
**Disease group**		
Asthma	258 (47.3 %)	3 (100 %)
COPD	288 (52.7 %)	0 (0 %)
**MMEF (% predicted)**	60.2 (53.6–65.5)	74.2 (72.3–74.4)
**MMEF Z-score**	−1.34 (−1.50 to −1.13)	−1.66 (−1.68 to −1.66)
**FEV**_**1**_**/FVC**	71.0 (67.0–75.0)	81.6 (80.5–85.3)
**FEV**_**1**_**/FVC Z-score**	−1.08 (−1.49 to −0.67)	−0.98 (−0.99 to −0.51)

Legend:

Values are reported as mean ± SD or median (Q1 to Q3) for continuous variables and n (%) for categorical variable.

**Abbreviations: FP,** False positive; **FN,** False negative; **BMI**, body mass index; **MMEF**, maximal mid-expiratory flow; **FEV**_**1**_**/FVC**, the ratio of the forced expiratory volume in the first second to the forced vital capacity.

The four MMEF% predicted cut-offs (i.e., 80, 70, 65 and 60) were evaluated using a regression model. Age was found to be a predictor for FP result across all cut-off ranges. People from Black, Asian, and minority ethnicity (BAME) were less likely to have FP results across all cut-off ranges. Males were more likely to have FP results than females across all cut-off ranges except 80 % predicted where no difference was found. Height was not found to be an independent predictor for FP using any of the cut-off ranges. Weight was a predictor for FP across all groups except the 60 % predicted group. Body mass index was a predictor for FP across all groups.

Age groups were analyzed across all % predicted groups except the 60 % where it was omitted due to the lack of FP in two age groups (i.e., <30 and 30–39 years). Older subjects were more likely to have a FP result compared to those <30 across all reported % predicted cut off groups. In Table 3, the odds ratios, 95 % confidence intervals, and p-value are provided for all characteristic predictors.

**Table 3:** The odds ratios (OR) of reporting false positives based on different % predicted values for MMEF.

The cut off order	80 %			70 %			65 %			60 %		
Characteristic	Event N	OR (95 % CI)	p-value	Event N	OR (95 % CI)	p-value	Event N	OR (95 % CI)	p-value	Event N	OR (95 % CI)	p-value
**Sex^**	837		0.25	546		**0.004**	392		< **0.001**	268		**0.003**
Female		–			–			–			–	
Male		1.10 (0.94–1.29)			1.32 (1.09–1.59)			1.45 (1.16–1.80)			1.49 (1.15–1.93)	
Age*	837	1.01 (1.01–1.02)	< **0.001**	546	1.03 (1.02–1.03)	< **0.001**	392	1.05 (1.04–1.06)	< **0.001**	268	1.08 (1.06–1.09)	< **0.001**
**BMI**^**#**^	837	1.03 (1.02–1.04)	< **0.001**	546	1.03 (1.02–1.04)	< **0.001**	392	1.03 (1.01–1.05)	**0.007**	268	1.03 (1.01–1.05)	< **0.001**
**Ethnicity**	837		**0.029**	546		< **0.001**	392		< **0.001**	268		**0.003**
White		–			–			–			–	
BAME		0.79 (0.63–0.98)			0.57 (0.43–0.75)			0.52 (0.36–0.72)			0.57 (0.37–0.83)	
Age Group*	837		< **0.001**	546		< **0.001**	392		< **0.001**			
<30		–			–			–				
30–39		0.67 (0.46–0.98)			0.95 (0.54–1.66)			5.87 (1.00–111)				
40–49		1.02 (0.74–1.43)			1.84 (1.16–2.99)			34.8 (7.54–618)				
50–59		0.93 (0.69–1.27)			1.78 (1.15–2.83)			31.7 (6.94–561)				
60+		1.23 (0.93–1.63)			3.05 (2.06–4.71)			73.2 (16.4–1288)				
**Height**	837	1.00 (1.00–1.01)	0.29	546	1.01 (1.00–1.02)	0.19	392	1.00 (0.99–1.01)	0.73	268	1.00 (0.99–1.01)	0.98
**Weight**	268	1.01 (1.00–1.01)	0.054	546	1.01 (1.01–1.01)	< **0.001**	392	1.01 (1.00–1.01)	**0.008**	837	1.01 (1.01–1.01)	< **0.001**

**Legend:** The false positive rates are reported based on the % predicted for MMEF. The Event N represent the number of false positive events from the total sample (n = 3396).The 60 % predicted age groups were omitted due to the lack of false positives in the reference group (<30) and 30–39 age group. * Adjusted for sex and BMI. **^** Adjusted for age and BMI. ^**#**^ Adjusted for age and sex.

**Abbreviations: BMI,** Body mass index; **OR**, Odds ratio; **CI**, Confidence interval; **BAME,** Black, Asian and Minority Ethnic.

## 4 Discussion

Overall, the use of a % predicted cut off for MMEF overestimates the presence of physiological abnormalities compared to deviation from the normal distribution using z-scores. The % predicted values for defining SAD has yielded high rates of abnormality in older population [20], which may reflect the aging lung rather than actual rate of ill health. In the current study, the LLN of % predicted in MMEF was inversely proportional to age. Evidence has suggested that MMEF is highly correlated with FEV_1_/FVC [7], and in this study we demonstrate that the FPs defined by MMEF using percent predicted had FEV_1_/FVC ratios within the normal range, supporting the likelihood of these being true FP results.

There is clear evidence that those older in age are more likely to be misclassified as having SAD based on arbitrary % predicted cut-off values compared to those classified by LLN based on z-score. Indeed, in population based studies greater age has been found to be a predictor of SAD when a fixed % predicted value was utilised [20,21], whereas a study that utilised LLN for MMEF in COPD patients did not find greater age to be a predictor for SAD [22].

Males were more likely to have FP rate using a % predicted of ≤70, despite being adjusted for age and BMI. The GLI dataset had more female (57.1 %) than male subjects (42.9 %) [18] which may have influenced the variability of % predicted values for males compared to females. Moreover, reduction of small airways function as measured by MMEF was previously reported to be higher in males than females [23]. However, in our model, environmental and conditional confounder such as smoking status and occupation were not accounted for which may limit the generalizability of our finding in sex differences. Moreover, this association appears to be small (70 % predicted: OR = 1.37; 95 % CI: 1.09–1.59) which warrants further research. This study used a specific cohort of patients with respiratory symptoms of asthma or COPD in a tertiary hospital in the UK, which limits the generalizability of the results.

Despite being a specific sample of patients, some limitations are found which may limit the generalizability of the findings. There may be sampling bias that limits as the cohort was limited to patients with asthma or COPD symptoms. Moreover, the data was collected from patients being treated in a tertiary hospital in the West Midlands of the UK which may have different environmental factor or treatment strategy compared to other areas.

If it is accepted that the small airways are dysfunctional in some patients with “early” asthma and COPD and that this is a warning or treatable trait, it would be critical to establish a robust evaluation and reporting criteria for MMEF. We suggest the use of z-scores in routine clinical practice as well as clinical trials, will provide more accurate and a deeper understanding of the presence or absence of physiological impairment in the small airways.

## 5 Conclusion

Small airways dysfunction is prevalent in chronic respiratory diseases.; However, utilising MMEF z-scores for defining abnormality avoids overestimating the burden of abnormality/disease. Z-scores can be easily obtained, interpreted and are more accurate than % predicted values. Therefore, it is suggested that this parameter should be utilised in future clinical trials and practice.

## Ethics approval and consent to participate

The study and all study activities were approved by the Health Research Authority (HRA IRAS number 274729; REC Reference: 20/HRA/0203).

## Availability of data and materials

Data will be made available on request.

## CRediT authorship contribution statement

**Mohammed A. Almeshari:** Conceptualization, Data curation, Formal analysis, Methodology, Project administration, Writing – original draft, Writing – review & editing, Visualization. **Nowaf Y. Alobaidi:** Conceptualization, Data curation, Formal analysis, Methodology, Writing – original draft, Writing – review & editing. **Elizabeth Sapey:** Conceptualization, Formal analysis, Methodology, Supervision, Writing – original draft, Writing – review & editing. **Robert A. Stockley:** Formal analysis, Methodology, Writing – original draft, Writing – review & editing. **James A. Stockley:** Data curation, Formal analysis, Writing – original draft, Writing – review & editing.

## Declaration of competing interest

The authors declare that they have no known competing financial interests or personal relationships that could have appeared to influence the work reported in this paper.

## List of Abbreviations

**COPD**: Chronic Obstructive Pulmonary Disease
**MMEF**: Maxima-Mid Expiratory Flow
**BMI**: Body Mass Index
**FP**: False Positive
**OR**: Odds Ratio
**BAME**: Black, Asian And Minority Ethnic
**FVC**: Forced Vital Capacity
**FEV**_**1**_: Forced Expired Volume In 1st Second
**FEF**_**27-75**_: Forced Expiratory Flow Rate Between The 25th And 75th Percentiles
**FN**: False Negative
**ARTP**: Association For Respiratory Technology And Physiology
**SR**: Standardised Residuals
**LLN**: Lower Limit Of Normal
**SAD**: Small Airways Dysfunction
